# Supplementary material for: Iterative improvement in the automatic modular design of robot swarms
Source: PeerJ Comput Sci. 2020 Dec 7;6:e322. doi: 10.7717/peerj-cs.322 (PMC7924708; doi:10.7717/peerj-cs.322)
Supplement: Supplemental Information 3 [file peerj-cs-06-322-s003.zip › argos3/doc/api/standalone/a00481_source.html]

ARGoS: plugins/robots/foot-bot/simulator/physx\_footbot\_model.h Source File


- Main Page
- Related Pages
- Namespaces
- Classes
- Files

- File List
- File Members

# plugins/robots/foot-bot/simulator/physx\_footbot\_model.h

Go to the documentation of this file.

```
00001 
00007 #ifndef PHYSX_FOOTBOT_MODEL_H
00008 #define PHYSX_FOOTBOT_MODEL_H
00009 
00010 namespace argos {
00011    class CPhysXEngine;
00012    class CPhysXFootBotModel;
00013    class CFootBotEntity;
00014 }
00015 
00016 #include <argos3/plugins/simulator/physics_engines/physx/physx_single_body_object_model.h>
00017 
00018 namespace argos {
00019 
00020    class CPhysXFootBotModel : public CPhysXSingleBodyObjectModel {
00021 
00022    public:
00023 
00024       CPhysXFootBotModel(CPhysXEngine& c_engine,
00025                          CFootBotEntity& c_entity);
00026 
00027       virtual void UpdateFromEntityStatus();
00028 
00029    private:
00030 
00031       CFootBotEntity& m_cFootBotEntity;
00032    };
00033 
00034 }
00035 
00036 #endif
```

---

Generated on 10 Jul 2018 for ARGoS by 
 1.6.1
